# Neurochemical and behavioral characterization of neuronal glutamate transporter EAAT3 heterozygous mice

**DOI:** 10.1186/s40659-017-0138-3

**Published:** 2017-09-19

**Authors:** Luis F. González, Francisca Henríquez-Belmar, Claudia Delgado-Acevedo, Marisol Cisternas-Olmedo, Gloria Arriagada, Ramón Sotomayor-Zárate, Dennis L. Murphy, Pablo R. Moya

**Affiliations:** 1 Laboratorio de Neuroquímica y Neurofarmacología, Centro de Neurobiología y Plasticidad Cerebral, Instituto de Fisiología, Facultad de Ciencias, Universidad de Valparaíso, Valparaíso, Chile; 2 Laboratorio de Neurogenética, Centro de Neurobiología y Plasticidad Cerebral, Instituto de Fisiología, Facultad de Ciencias, Universidad de Valparaíso, Valparaíso, Chile; 3 Núcleo Milenio Nu-MIND Biology of Neuropsychiatric Disorders, Valparaíso, Chile; 4 Centro Interdisciplinario de Neurociencias de Valparaíso CINV, Valparaíso, Chile; 5 Departamento de Ciencias Biologicas, Facultad de Ciencias Biologicas, Universidad Andres Bello, Viña del Mar, Chile; 6 Laboratory of Clinical Science, National Institute of Mental Health, NIH, Bethesda, MD 20892 USA

**Keywords:** EAAT3, *SLC1A1*, Neuronal glutamate transporter, Obsessive–compulsive disorder

## Abstract

**Background:**

Obsessive–compulsive disorder (OCD) is a severe neuropsychiatric condition affecting 1–3% of the worldwide population. OCD has a strong genetic component, and the *SLC1A1* gene that encodes neuronal glutamate transporter EAAT3 is a strong candidate for this disorder. To evaluate the impact of reduced EAAT3 expression in vivo, we studied male EAAT3 heterozygous and wild-type littermate mice using a battery of behavioral paradigms relevant to anxiety (open field test, elevated plus maze) and compulsivity (marble burying), as well as locomotor activity induced by amphetamine. Using high-performance liquid chromatography, we also determined tissue neurotransmitter levels in cortex, striatum and thalamus—brain areas that are relevant to OCD.

**Results:**

Compared to wild-type littermates, EAAT3 heterozygous male mice have unaltered baseline anxiety-like, compulsive-like behavior and locomotor activity. Administration of acute amphetamine (5 mg/kg intraperitoneally) increased locomotion with no differences across genotypes. Tissue levels of glutamate, GABA, dopamine and serotonin did not vary between EAAT3 heterozygous and wild-type mice.

**Conclusions:**

Our results indicate that reduced EAAT3 expression does not impact neurotransmitter content in the corticostriatal circuit nor alter anxiety or compulsive-like behaviors.

**Electronic supplementary material:**

The online version of this article (doi:10.1186/s40659-017-0138-3) contains supplementary material, which is available to authorized users.

## Background

Obsessive–compulsive disorder (OCD) is a persistent, disabling neuropsychiatric condition affecting 1–3% of the worldwide population. OCD is characterized by persistent intrusive thoughts (obsessions), repetitive ritualistic behaviors (compulsions) and excessive anxiety [1]. Family, twin and case–control studies have shown that genetic factors play a major role in OCD (for a review, see [2]).

Altered glutamatergic neurotransmission has been postulated in the etiology of OCD. The glutamatergic hypothesis has accumulated evidence from neuroimaging studies [3, 4], animal models with altered glutamatergic neurotransmission exhibiting compulsive-like behaviors [5–7] and reports of beneficial effects of anti-glutamatergic agents on treatment-resistant OCD [8]. Genetic linkage and association studies have implicated glutamate system genes in OCD; among them, the most consistent candidate gene in OCD is *SLC1A1* (solute carrier, family 1, member 1) gene [1, 9–15]. *SLC1A1* encodes for the neuronal excitatory amino acid transporter EAAT3, with reported roles in controlling glutamate spillover which affects extrasynaptic NMDA and metabotropic glutamate receptors activity [16, 17].

Mice lacking EAAT3 (KO) were first reported 20 years ago; the original report showed that EAAT3 KO mice have reduced locomotor activity, but no neurological or cognitive impairments [18]. Given its role in cysteine uptake, EAAT3 KO mice also have neuronal glutathione depletion and greatly enhanced susceptibility to oxidative damage [18, 19]. No comprehensive behavioral assessment, in particular OCD-related behaviors was available until a very recent report showing that EAAT3 KO mice have unaltered baseline anxiety-like or compulsive-like behaviors, and reduced sensitivity to behavioral effects induced by amphetamine or SKF-38393, a dopamine D_1_ receptor agonist [20].

Animal models with diminished, rather than absent gene expression are of clinical relevance, because they might better reflect the impact of human polymorphisms affecting protein levels, where complete loss of expression is very rare [21]. Therefore, to assess the role of reduced EAAT3 expression, we evaluated the neurochemical and behavioral alterations in EAAT3 heterozygous (HET) mice.

## Methods

### Animals

Male mice carrying a targeted knockout *Slc1a1* gene were generated at the NIMH Transgenic Core Facility using a construct obtained from Knockout Mouse Project Consortium (KOMP) (clone ID: PG00093 Z_1_807) under a standard protocol. Briefly, linearized construct was electroporated in embryonic stem (ES) cells; after selection screening, targeted ES cells were injected into C57BL/6N blastocysts and chimeric mice were bred with C57BL/6J to test for germ line transmission and to establish the targeted lines. The official designation of these mice is B6-Slc1a1^*tm1a(KOMP)Wtsi*^. By the time of experimental procedures, 10 crosses onto C57BL/6J have been made. Three to five months old EAAT3 HET and wild-type (WT) littermates were used in all experiments. Mice were housed in groups of 3–5 per cage on a 12-h light:12-h dark cycle (lights on at 07:00) with food and water ad libitum in a facility approved by the Institutional Animal Welfare Committee. Efforts were made to minimize the number of animals used and their suffering.

Genotyping was performed using genomic DNA from 0.5 cm tail biopsies. DNA extraction was performed by standard procedures. LoxP primers flanking downstream LoxP site generate at 210 bp band in knockout allele, and 185 bp in wild-type allele: LoxP-For (5′-ACCCAATTTCACACCCTCCTCAGC-3′); LoxP-Rev (5′-GATTTCGTTTCTACCTCGGGCCTA-3′). 1a1-En2 primers targeting the initial 3′ portion of the cassette interrupting transcription generate a 500 bp in the KO allele and no band in the WT allele: 1a1En2-for (5′-TGGCTCGGGTTTCTCCTAGCTGGT-3′); 1a1-en2-Rev (5′-CCAACTGACCTTGGGCAAGAACAT-3′). PCR reactions used 0.4 µM primer concentration, and were performed using SapphireAmp Fast PCR master mix (Takara, Shiga, Japan) and the following thermal cycle: 95 °C 10 min; [94 °C 30 s, 57 °C 30 s, 72 °C 30 s] × 30 cycles; 72 °C 10 min. Amplicons were run in a 2% agarose electrophoresis in TBE buffer and visualized in UV light for analysis.

Quantitative reverse transcription and real-time polymerase chain reaction (qRT-PCR). EAAT3 HET and WT mice were killed by cervical dislocation; brains were removed immediately, striatum regions were dissected on a glass-plate set in ice, and stored at −80 °C until use. Total RNA was extracted from homogenized striata by sonication and using RNAeasy Mini Kit (Qiagen, Carlsbad, CA, USA), and eluted with RNase-free water. RNA was quantified using a nanospetrofotometer (Nanodrop ND-1000, Thermo Scientific, Waltham, MA, USA) and integrity confirmed by non-denaturant horizontal electrophoresis in agarose during 1 h at 120 V. Reverse transcription was performed using 10 ng of total RNA using the Takara prime script Reagent kit containing genomic DNA elimination step (Clontech, Mountain View, CA, USA). *Slc1a1* (EAAT3), *Slc1a2* (GLT-1a, the major isoform in brain) and *Slc1a3* (GLAST) mRNA levels were measured with respect to the housekeeping gene Hypoxanthine-guanine phosphoribosyltransferase 1 (Hprt1) mRNA using the following primers: GCTACATGCCGATTGGCATT and TACCCAAGGCAAAGCGGAAA for Slc1a1; TGTCTATGCCGCACACAACT and TCCTCAACACTGCAGTCAGC for Slc1a2; GGATGGAAAGATTCCAGCAA and GCTGACGGTGAGTAGCACAA for Slc1a3; CAAACTTTGCTTTCCCTGGT and TCTGGCCTGTATCCAACACTTC for Hprt1. Real-time PCR was performed with using Brilliant III Ultra-Fast SYBR Green qRT-PCR Master Mix (Santa Clara, CA, USA) in a 10 µl reaction volume with primers at 0.2 µM final concentration, using a CFX96 Connect System (Bio-Rad, Hercules, CA, USA). Amplification protocol consisted in 40 cycles as follows: 10 s of denaturation at 95 °C; 45 s of annealing at 58 °C; 15 s of elongation at 72 °C. All reactions were performed in triplicate; expression changes were analyzed as previously described [22].

### EAAT3 Western Blot

EAAT3 HET and WT mice were killed by cervical dislocation; brains were removed immediately and striatum was dissected on a glass-plate set in ice, and stored at −80 °C until use. Striatum samples were homogenized in ice-cold RIPA buffer with plastic homogenizer for minitubes. Homogenates were centrifuged at 13,000 rpm at 4 °C for 20 min; pellet was discarded, and supernatant protein concentration was calculated using Bradford method. Samples in loading buffer (Tris–HCl 125 mM pH 6.8; glycerol 20%, SDS 4%, β-mercaptoethanol 2, 0.02% of bromophenol blue) were denatured by heating at 70 °C for 8 min. 30 µg protein were loaded in a polyacrylamide/SDS 8% gel and separated by electrophoresis in running buffer 1× (Tris 2.5 mM, glycine 19.2 mM, SDS 0.01%) by 90 min at 100 V, and then transferred to a nitrocellulose membrane for 90 min at 450 mA in transfer buffer (Tris 2.5 mM, glycine 19.2 mM, SDS 0.01, 20% metanol); transfer was verified using Ponceau red staining. Membranes were washed in TBS-Tween (T-TBS) 1× for 5 min, and then blocked using 5% skim milk solution in PBS for 1 h. Primary monoclonal antibody anti-EAAT3 (AB124802, Abcam, Cambridge, UK) was dissolved in blocking solution and incubated overnight at 4 °C. Next day, membranes were washed three times with T-TBS 0.1% for 5 min. Secondary antibody goat anti-rabbit (HRP) (ab205718, Abcam) was incubated for 1 h; membranes were then washed three times with T-TBS. Membranes were loaded with Pierce ECL chemiluminescent substrate (ThermoFisher Scientific, Waltham, MA, USA) for 1 min and revealed for 12 min. Beta-actin antibody (ab8227, Abcam) was used as normalizer. Images were analyzed by ImageJ software (NIH, Bethesda, MD, USA) as described previously [22].

### Behavioral analyses

Mice in their home cages were acclimated to the behavioral room at least 1 h before analyses. Tests were performed once per day and performed sequentially: Open field, elevated plus maze, marble burying and locomotor activity. All experiments were performed in dim light set at 20 *lux*; 12–13 mice per group were used.

Open field test: each animal was individually placed in a Plexiglass box (40 × 40 × 35 cm) and allowed to freely explore for 5 min. Behavior including time and frequency in center (20 × 20 cm) were recorded and analyzed using Noldus Ethovision XT (Noldus Information Technology, Leesburg, VA, USA) as previously described [23].

Elevated plus maze test: after acclimation, each mouse was placed in an elevated plus maze (30 cm arm, 46 cm height closed arms) and allowed to freely explore for 5 min. Time and number of entrances to closed and open arms were recorded and analyzed using Ethovision XT.

Marble burying test: after acclimation, mice were individually placed in a clean home cage containing 4–5 cm bedding material; 15 dark glass marbles (1.5 cm diameter) were placed in a 3 × 5 distribution across the surface. After 15 min, mice were removed and the number of marbles buried at least 2/3 of its surface was recorded by an experimenter blind to the animal genotype.

Locomotor activity: baseline horizontal activity was monitored over 30 min in the open field arena. Mice received then an intraperitoneal (i.p.) saline injection and were monitored for other 30 min. Next, a single dose of amphetamine (5 mg/kg, i.p.) was administered and activity was recorded for additional 60 min. Videos were analyzed using Noldus Ethovision XT.

### Analysis of brain region neurotransmitter and metabolites

In a separate cohort, EAAT3 HET and WT mice were killed by cervical dislocation. Brains were removed immediately and dissected on a glass-plate set in ice, weighed in an analytical balance and stored at −80 °C until use, as previously described [24]. Samples were homogenized according to a protocol modified from Cruz et al. [25]. Briefly, each brain tissue was collected in 400 µL of 0.2 M perchloric acid and then homogenized in a sonicator. The homogenate was centrifuged at 12,000×*g* for 15 min at 4 °C (model Z233MK-2, Hermle LaborTechnik GmbH, Wehingen, Germany) and the resultant supernatant was filtered (0.2 µm HPLC Syringe Filters disposable filter PTFE, model EW-32816-26, Cole-Parmer Instrument Company, USA). The filtered supernatants were injected into a HPLC coupled to electrochemical detection (for determination of DA, DOPAC, 5-HT and 5-HIAA contents) and fluorometric detection (GLU and GABA).

DA, 5-HT, DOPAC and 5-HIAA quantifications: experimental conditions were as described previously [25–27]. Ten microliters of each cleaned supernatant was injected to the HPLC system with the following setting: A isocratic pump, (model PU-2080 Plus, Jasco Co. Ltd., Tokyo, Japan), a UniJet microbore column (MF-8912, BAS, West Lafayette, IN, USA) and an electrochemical detector (set at 650 mV, 0.5 nA; model LC-4C, BAS, West Lafayette, IN, USA). The mobile phase, containing 0.1 M NaH_2_PO_4_, 1.0 mM 1-octanesulfonic acid, 0.27 mM EDTA and 4.0% (^v^/_v_) CH_3_CN (pH adjusted to 2.6) was pumped at a flow rate of 0.1 mL/min. DA, DOPAC, 5-HT and 5-HIAA levels were assessed by comparing the respective peak area and elution time of the sample with a reference standard and the quantification was performed using a calibration curve for each neurotransmitter (Program ChromPass, Jasco Co. Ltd., Tokyo, Japan).

GLU and GABA quantifications: experimental conditions were as described previously [28, 29]. Briefly, 20 μL of each cleaned supernatant was mixed with 4 μL of borate buffer (pH 10.8), and then the mixture was derivatized by adding 4 μL of fluorogenic reagent (20 mg of orthophthaldehyde and 10 μL of β-mercaptoethanol in 5 mL of ethanol). At 90 s after pre-column derivatization, samples were injected into an HPLC system with the following configuration: an isocratic pump (Jasco Co. Ltd), a C-18 reverse phase column (Kromasil; Eka Chemicals, Bohus, Sweden), and a fluorescence detector configured for an excitation wavelength of 340 nm and an emission wavelength of 450 nm (Jasco Co. Ltd). A mobile phase containing 0.1 M NaH_2_PO_4_ and 14.5% (^v^/_v_) CH_3_CN (pH 5.7) was pumped for 4 min. The flow rate of the mobile phase was set at 1.0 mL/min.

### Statistical analyses

For each experiment, data were analyzed using t-tests or two-way (genotype x drug condition) analyses of variance. Significant interactions were followed by *post-hoc* comparisons between genotypes or between drug conditions using t-test or Tukey HSD pairwise comparisons. Significance was set on P < 0.05. Data are presented as mean ± SEM.

## Results

**Fig. 1:**
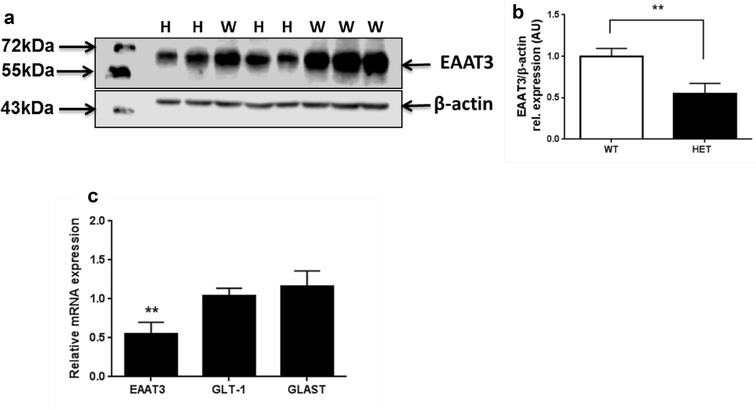
Reduced EAAT3 expression in EAAT3 heterozygous mice compared to wild-type littermates. **a** Representative Western Blot showing reduced EAAT3 protein expression in HET mice (H lanes) compared to WT mice (W lanes). **b** Graph bar of blot quantification; unpaired t-test P = 0.0076, n = 4 per group. **c** Real time qPCR detected significantly reduced EAAT3 and unaltered GLT-1 and GLAST mRNA levels in EAAT3 HET mice compared to WT littermates. Unpaired t-test with Welch´s correction (against WT mRNA levels): EAAT3 P = 0.0025; GLT-1 P = 0.9467, N.S.; GLAST P = 0.3980, N.S. n = 4 samples per group, each sample run in triplicate. ** indicates P<0.01

We first measured EAAT3 protein in striatal protein preparations from EAAT3 HET and WT littermates. As shown in Fig. 1a, EAAT3 HET mice showed 46% reduction in protein levels compared to WT (P < 0.0076, n = 4 mice per group). mRNA levels were also evaluated for *Slc1a1* (EAAT3) and other glutamate transporters such as *Slc1a2* (GLT1) and *Slc1a3* (GLAST). As shown in Fig. 1b, qPCR measurements indicate a downregulation near to a half of *Slc1a1* in the EAAT3 HET group compared to expression levels in WT group (0.0553 ± 0.0743). *Slc1a2* and *Slc1a3* mRNA expression values in EAAT3 HET group were not statistically different compared to WT control group (1.039 ± 0.0994 and 1.158 ± 0.1010, respectively, N.S.). Uncropped blot is available in Additional file 1, as well as representative control blot assessing EAAT3 expression in different tissues from WT mice.

**Fig. 2:**
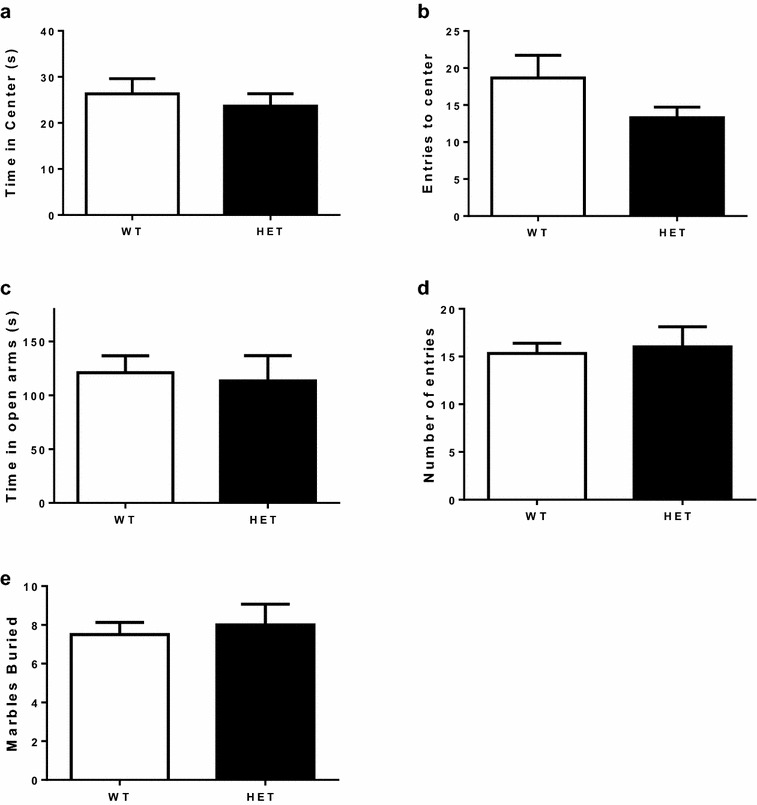
No differences in anxiety-like or compulsive-like behaviors between EAAT3 heterozygous mice and wild-type littermates. **a** Time spent in center of open field arena; unpaired t-test P = 0.5272, N.S. **b** Number of entries to the center of arena (counts); unpaired t-test P = 0.1154, N.S. **c** Time spent in open arms of the elevated plus maze; unpaired t-test P = 0.7840, N.S. **d** Number of entries (counts) to open arms; unpaired t-test P = 0.7599, N.S. **e** Number of buried marbles; unpaired t-test P = 0.6929. Data represent the mean ± SEM, 12–13 animals per group

Then, we evaluated if reduced EAAT3 expression in EAAT3 HET mice might impact anxiety-like behavior by using the open field test and the elevated plus maze, two well-established behavioral paradigms,. No differences were found in the open field test between EAAT3 HET and WT littermates in time spent in center (Fig. 2a; 26.3 ± 3.3 vs 23.6 ± 2.7 s, respectively, N.S.; data expressed as mean ± SEM) or in the number of visits to center area (Fig. 2b; 18.7 ± 3.1 vs 12.7 ± 1.4, respectively, N.S). Similarly, as shown in Fig. 2c, we found no differences in the elevated plus maze in EAAT3 HET mice compared to WT littermates in time spent in open arms (113.4 ± 23.4 vs 121.0 ± 15.7 s, respectively, N.S.) or in the number of entries to open arms (Fig. 2d; 15.3 ± 1.0 vs 16.0 ± 2.1, respectively, N.S.).

Next, as a proxy of compulsive behavior, we used the marble burying test. As depicted in Fig. 2e, no differences were found between EAAT3 HET mice compared to WT littermates. The numbers of buried marbles were as follows: EAAT3 HET male = 8.0 ± 1.1; WT = 7.5 ± 0.6, N.S.

**Fig. 3:**
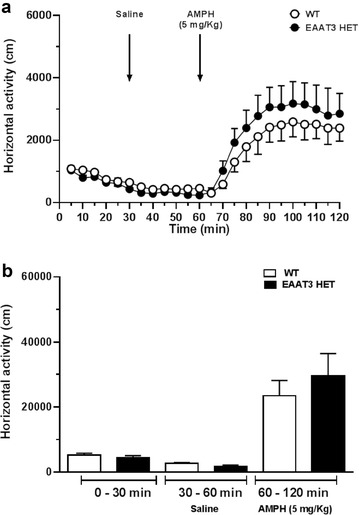
No changes in baseline or amphetamine-induced locomotor activity in EAAT heterozygous mice compared to wild-type littermates. **a** Locomotor activity baseline and induced by a single dose of amphetamine 5 mg/kg (i.p.). **b** Cumulative locomotor activity. Two-way ANOVA (genotype x drug) interaction P = 0.4087, N.S. Data represent the mean ± SEM, 6–7 animals per group

Since EAAT3 has been shown to regulate dopaminergic neuron activity, we evaluated if EAAT3 HET mice would have affected sensitivity to amphetamine-induced hyperlocomotion. Figure 3 shows that baseline horizontal locomotor activity was unaltered in EAAT3 HET mice compared to WT littermates over a 30 min observation period. No changes were found upon saline administration. Then, a single dose of amphetamine (5 mg/kg i.p.) was administered; increased locomotion was observed in both groups, being unaffected by genotype (ANOVA drug x genotype N.S., P = 0.4087; drug effect F(1,16); P < 0.0001) .

From a separate cohort of animals, we measured neurotransmitter alterations in prefrontal cortex, striatum and thalamus, areas belonging to the cortical-striatal-thalamo cortical loop that has been postulated to be altered in OCD [17]. As shown in Table 1, we found no changes in glutamate or GABA tissue content in any of the tested regions; monoamines and their metabolites were also unaltered.

**Table 1:** Neurotransmitter tissue contents in EAAT3 HET and WT littermates determined by HPLC coupled to electrochemical (monoamines) and fluorometric (aminoacids) detection

*Slc1a1* genotype	Glutamate (pg/mg tissue)	GABA (ng/mg tissue)	5-HT (pg/mg tissue)	5-HIAA (pg/mg tissue)	Dopamine (pg/mg tissue)	DOPAC (pg/mg tissue)
Prefrontal cortex						
WT	73.1 ± 9.0 n = 7	4605 ± 714.8 n = 6	525.4 ± 30.5 n = 7	581.3 ± 45.3 n = 7	35.2 ± 7.7 n = 6	N.D
HET	67.7 ± 5.9 n = 7	4923 ± 338 n = 6	466.4 ± 37.2 n = 7	520.4 ± 33.0 n = 7	55 ± 12.9 n = 6	N.D
t-test	N.S.	N.S.	N.S.	N.S.	N.S.	
Striatum						
WT	100.9 ± 10.0 n = 7	6973 ± 463.3 n = 7	473.9 ± 51.5 n = 7	667.6 ± 76.8 n = 7	10,631 ± 1147 n = 6	2126 ± 370.6 n = 6
HET	90.1 ± 11.4 n = 7	7658 ± 1372 n = 7	512 ± 51.8 n = 7	792.9 ± 72.3 n = 7	9593 ± 1296 n = 7	1818 ± 174.8 n = 7
t-test	N.S.	N.S.	N.S.	N.S.	N.S.	N.S.
Thalamus						
WT	67.4 ± 7.6 n = 7	13,449 ± 1714 n = 7	720.9 ± 64.5 n = 6	2078 ± 117.8 n = 5	227.9 ± 27.2 n = 7	55.3 ± 9.4 n = 6
HET	49.6 ± 9.1 n = 7	11,785 ± 1965 n = 7	618.5 ± 113.4 n = 7	1821 ± 201.3 n = 7	183.1 ± 22.2 n = 7	72.2 ± 11.7 n = 6
t-test	N.S.	N.S.	N.S.	N.S.	N.S.	N.S.

Please see “Methods” section for further details. Data are expressed as mean ± SEM

## Discussion

EAAT3 plays multiple roles in regulating neuronal function that might be relevant for OCD. It is prominently expressed in the cortical-striatal-thalamic-cortical circuit implicated in OCD [30]. EAAT3 is expressed in glutamatergic, GABAergic and dopaminergic neurons [31]. It has been shown that EAAT3 can regulate glutamate clearance, synaptic plasticity and glutamate spillover in excitatory synapses [32, 33]. Our results show that EAAT3 HET mice are unaffected at baseline behavioral levels in anxiety-like or compulsive-like behavior. Tissue content analyses showed no significant changes in glutamate, GABA or monoamine neurotransmitter levels, in any of the evaluated brain regions comprising the CSTC circuit that is dysregulated in OCD. The lack of impact of reduced EAAT3 levels (and plausibly its function) might indicate that protein expression from a single allele copy would suffice to account for EAAT3 activity, without altering the parameters determined here, i.e. tissue neurotransmitter system, anxiety-like and compulsive-like behavior, and amphetamine-induced locomotion. Alternatively, this could be due to compensatory upregulation of other glutamate transporters such as GLT1 and GLAST. We found that *Slc1a2* (GLT-1) and *Slc1a3* (GLAST) mRNA levels did not significantly vary between EAAT3 HET and WT mice; this is in agreement with previous findings showing that EAAT3 full deletion did not upregulate other glutamate transporters in EAAT3 KO mice [18]. We cannot rule out, however, that significant protein changes might occur for these two glutamate transporters in our EAAT3 HET mice, which requires further investigation. Indeed, it has been shown that the selective removal of astrocytic GLT-1 triggers repetitive behaviors such as grooming and tic-like movements, highlighting the importance of other glutamate transporters in the pathophysiology of OCD beyond EAAT3 [34]. In addition, compensatory changes on other critical components of glutamatergic transmission machinery including AMPA and NDMA receptors and their subunit composition could account for the negative findings. In this regard, it has been shown that, in the hippocampus, EAAT3 regulates activation of NMDA receptors by regulating glutamate spillover and that the activation is dependent on NR2B containing NMDA receptors [32]. The blockade of EAAT3 function, which increases local glutamate concentration, leads to activation of perisynaptic NR2B containing NMDA receptors which, in turn, lead to changes in the trafficking of AMPA receptors [16, 35].

When expressed in GABAergic neurons, EAAT3 provides the precursor for the synthesis of GABA [32]. In this regard, we found no alterations in GABA levels in striatum, region containing a high number of GABAergic medium spiny neurons, suggesting that partial deletion of EAAT3 did not impact tissue levels of this neurotransmitter. However, we cannot rule out that alterations in EAAT3 expression might impact the strength of GABAergic neurotransmission; such possibility needs to be tested thoroughly using approaches like in vivo microdialysis or electrophysiological recordings.

The majority of glutamate is cleared from extracellular space by glial Slc1a2/GLT-1 and Slc1a3/GLAST; it has been estimated that over 80% of glutamate uptake is due to the activity of these transporters [30]. Thus, unaffected glutamate tissue levels were indeed expected since the contribution of EAAT3 to the overall glutamate clearance is minimal.

EAAT3 is expressed in dopaminergic neurons from mesolimbic and nigrostriatal pathways [30, 36]. Recently, Zike and colleagues showed that EAAT3 KO mice have reduced sensitivity to amphetamine effect on locomotor activity [20]. In this regard, we found no significant alterations in amphetamine-induced locomotor activity in EAAT3 HET mice. Correspondingly, levels of DA and DOPAC, its metabolite, were unaltered in the brain regions tested in this report, although we did not measure midbrain areas such as ventral tegmental area or substantia nigra.

Early reports showed EAAT3 KO mice exhibit several neuronal defects largely attributed to oxidative stress due to impaired glutathione synthesis but no OCD-related behaviors [18, 19]. Human *SLC1A1* homozygous loss-of-function mutations are extremely rare in humans, and cause the renal condition dicarboxylic aminoaciduria [37] which is also present in EAAT3 KO mice [18]. Rather, common polymorphisms can affect gene expression in less dramatic ways than the loss-of-function represented in a knockout mouse model. As such, we aimed to characterize an intermediate reduction of EAAT3 expression, which could be relevant to the study of neurobiological basis of this disorder [21].

*SLC1A1* is one of the most consistently associated genes in OCD. The majority of findings cluster in the 3′ region, and in particular most evidence points to the rs301430C allele [1, 6, 15]. Both in cell assays and *post*-*mortem* brain tissue, this allele is associated with increased *SLC1A1* expression, suggesting that increased, rather than decreased expression contributes to OCD susceptibility [1, 35]. Therefore, the lack of significant changes in EAAT3 HET mice showed here is consistent with this notion; overexpression EAAT3 experiments are needed to test this hypothesis.

## Conclusions

Our results indicate that a *Slc1a1* heterozygosis in mice does not alter anxiety- or compulsive-like behaviors evaluated in this work, nor tissue neurotransmitter levels in the cortex, striatum and thalamus, comprising the brain circuit proposed to be altered in OCD. Our data are in line with a recent report [20] finding that EAAT3 KO mice have unaltered spontaneous OCD relevant behaviors.

## Additional file

**Additional file 1.** Uncropped blots for EAAT3 protein determinations.
