# Supplementary figures and images for: Neurochemical and behavioral characterization of neuronal glutamate transporter EAAT3 heterozygous mice
Source: Biol Res. 2017 Sep 19;50:29. doi: 10.1186/s40659-017-0138-3 (PMC5605982; doi:10.1186/s40659-017-0138-3)

Supplementary Figure
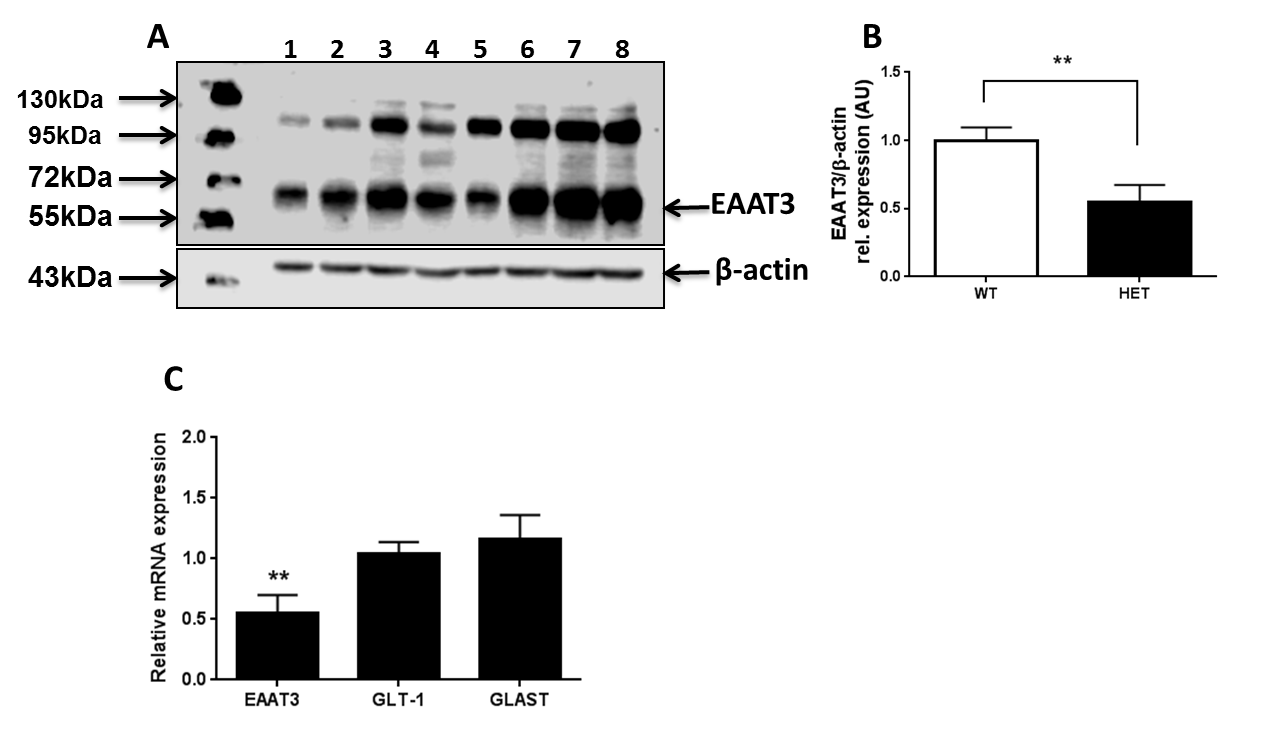


Uncropped blots for EAAT3 protein determinations.

Supplement: Supplementary file 1 — Additional file 1. Uncropped blots for EAAT3 protein determinations. [file 40659_2017_138_MOESM1_ESM.docx]
